# Crosstalk among long non-coding RNA, tumor-associated macrophages and small extracellular vesicles in tumorigenesis and dissemination

**DOI:** 10.3389/fonc.2022.1008856

**Published:** 2022-10-03

**Authors:** Li-jie Zhang, Feng Chen, Xiao-ru Liang, Murugavel Ponnusamy, Hao Qin, Zhi-juan Lin

**Affiliations:** ^1^ Key Lab for Immunology in Universities of Shandong Province, School of Basic Medical Sciences, Weifang Medical University, Weifang, China; ^2^ Department of General Surgery, Weifang Traditional Chinese Hospital, Weifang, China; ^3^ Institute for Translational Medicine, Qingdao University, Qingdao, China; ^4^ Department of Public Health, Weifang Medical University, Weifang, China

**Keywords:** lncRNA, sEV, TAMs, tumor microenvironment, tumorigenesis

## Abstract

Long noncoding RNAs (lncRNAs), which lack protein-coding ability, can regulate cancer cell growth, proliferation, invasion, and metastasis. Tumor-associated macrophages (TAMs) are key components of the tumor microenvironment that have a significant impact on cancer progression. Small extracellular vesicles (sEV) are crucial mediators of intercellular communications. Cancer cell and macrophage-derived sEV can carry lncRNAs that influence the onset and progression of cancer. Dysregulation of lncRNAs, TAMs, and sEV is widely observed in tumors which makes them valuable targets for cancer immunotherapy. In this review, we summarize current updates on the interactions among sEV, lncRNAs, and TAMs in tumors and provide new perspectives on cancer diagnosis and treatment.

## 1 Introduction

Non-coding RNAs (ncRNAs) are a class of RNA molecules that cannot encode proteins and are different from messenger RNA (mRNA) in structure and function (1). Although ncRNAs have been regarded as “junk RNA” and “transcriptional noise” in the past, the development of high-throughput sequencing has revealed the importance of ncRNAs in both physiological and pathological processes, including cell cycle, cell differentiation, and tumorigenesis (2–5). Based on their functions and sizes, ncRNAs are mainly divided into short ncRNAs, long ncRNAs (lncRNAs), and circular RNAs (circRNAs) (3, 6). LncRNAs are defined as transcripts longer than 200 nucleotides without protein coding abilities (7). They are important contributors to tumor growth, proliferation, invasion, and metastasis due to their ability to regulate the tumor microenvironment (TME) (8–10).

**Figure 1:**
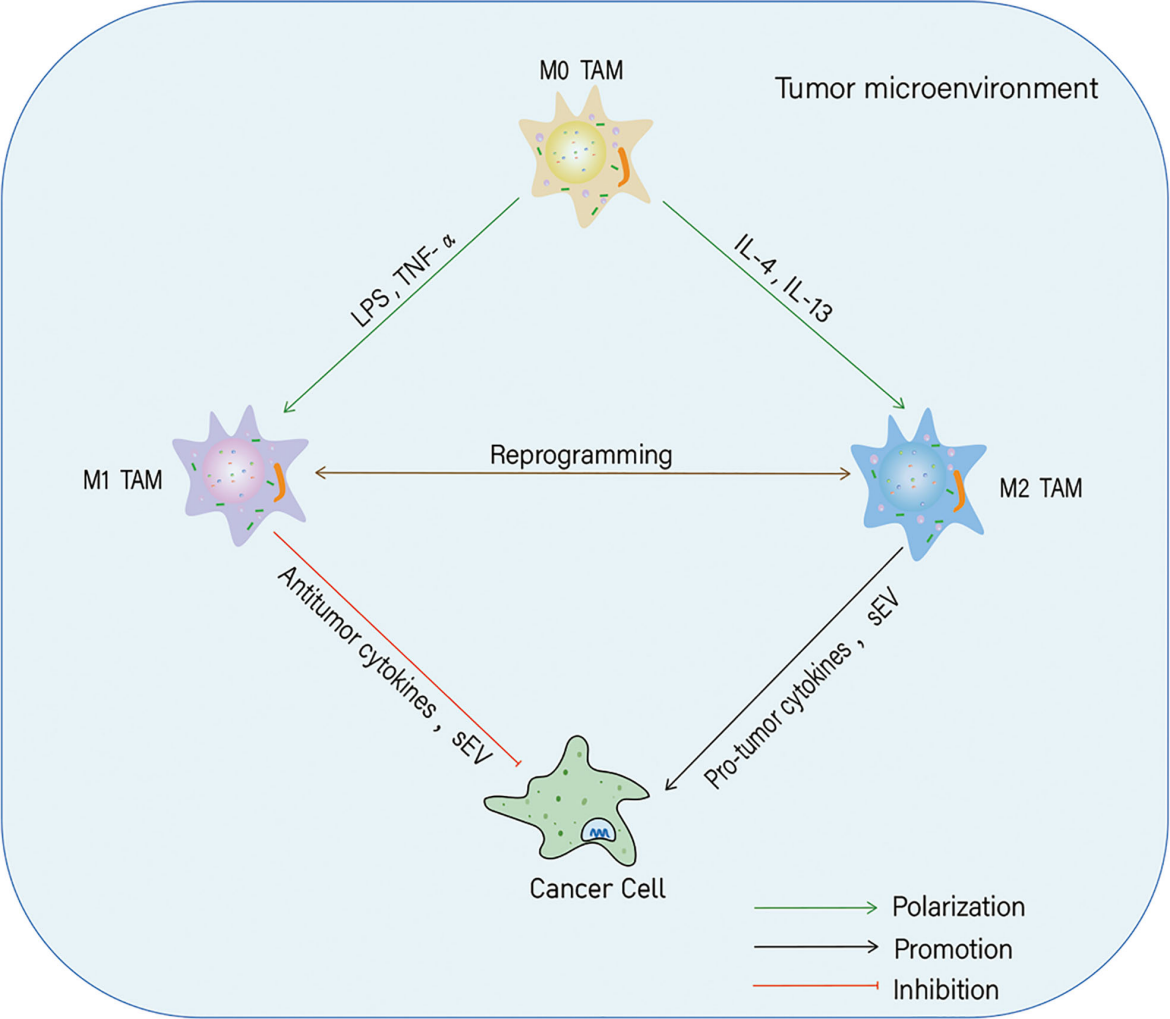
Polarization of tumor-associated macrophages (TAMs) and their effects on cancer cells. In the tumor microenvironment, macrophages can polarize into M1 and M2 phenotypes in response to different stimuli. Both M1-likeTAMs and M2-likeTAMs can secrete cytokines and small extracellular vesicles (sEV) to inhibit or promote tumor progression.

TME is a highly complex and dynamic network of cells, composed of immune, cancer, endothelial, and stromal cells, and blood vessels, fibroblasts, and the extracellular matrix and our understanding of the TME is continuously evolving (11). Among these components, macrophages, a class of immune cells characterized by diversity and plasticity, play a crucial role in the immune response (12). According to their activation status and function, macrophages are divided into M1 (classically activated) and M2 (alternatively activated) phenotypes (13). Macrophages differentiate into the M1 phenotype, when induced by lipopolysaccharide (LPS) and tumor necrosis factor-α (TNF-α) and have pro-inflammatory and antitumor functions. In contrast, interleukin (IL)-4 and IL-13 can induce macrophages to polarize towards the M2 phenotype, which plays anti-inflammatory and pro-tumorigenic roles (14). The plasticity of macrophages allows them to switch from one phenotype to another (15–17). Tumor-associated macrophages (TAMs) are major participants in TME formation and are abundantly found in a variety of tumors (18). The recruitment, polarization, and phenotypic transition of TAMs can regulate cancer occurrence and progression by modulating tumor metabolism and inducing cell proliferation, invasion, metastasis, drug resistance, and immune evasion (19–21). The polarization is affected by many factors, one of which are small extracellular vesicles (sEV) (**Figure 1**).

The sEV are secreted by various cells and have emerged as vital players in intercellular information exchange by transporting intracellular components such as proteins, nucleic acids (both RNA and DNA), and lipids (22, 23). sEV can alter the physiological state of recipient cells *via* different mechanisms. For example, sEV can directly attach to recipient cells through their surface molecules *via* receptor-ligand interactions (24, 25) or fuse with the target cell membrane to transport their contents into the cytosol (8, 26). sEV are derived from tumor cells and other cells of the TME, including fibroblasts, endothelial and immune cells, and have different signatures based on their origin (27). The sEV in the TME influence multiple tumorigenic behaviors such as invasion, metastasis, and angiogenesis (28).

LncRNAs derived from tumor cells and TAMs are selectively packaged into sEV that function as messengers for intercellular communication in the TME. The sEV carrying lncRNAs can regulate cancer onset and progression by affecting the recruitment, polarization, and phenotypic transition of TAMs (13). TAMs exert an immune regulatory role in the development of tumors by secreting various cytokines and effector molecules (6). In tumors, abnormally expressed lncRNAs, TAMs, and sEV can not only be used as diagnostic and prognostic markers but also as potential targets for cancer therapy. Only a few studies have explored the relationship between these three factors in cancer. Therefore, in this review, we summarize current updates to clarify the interactions among lncRNAs, TAMs, and sEV, and analyze the possible mechanisms involved in these interactions during the development and progression of cancer.

## 2 LncRNAs regulate TAMs and influence tumorigenesis and progression

LncRNAs play a vital role in the occurrence, development, and metastasis of cancers and influence tumor initiation and development by affecting the TME. In the TME, TAMs are one of the most abundant cells that are primarily responsible for pro-tumoral processes and immune evasion/suppression (29) and the lncRNAs are involved in their regulation (**Table 1**) (14, 57). While some lncRNAs can induce macrophage recruitment, polarization, and M1/M2 phenotypic transition to accelerate cancer progression others inhibit tumor development by regulating TAMs. However, the regulatory functions of lncRNAs in TAMs may depend on cancer cell type, tumor stage, and TME. In this section, we discuss the bilateral/dual effects of lncRNAs on TAMs and the underlying mechanisms that regulate the paradoxical functions of lncRNAs.

**Table 1:** The effects of lncRNAs on TAMs.

LncRNA	Tumor types	The effects on TAMs and mechanisms	reference
PCAT6	CCA	PCAT6 induces M2 polarization of macrophages via modulating miR-326 and RhoA-ROCK signaling	(30)
GNAS-AS1	ER^+^-BC	GNAS-AS1 facilitates M2 polarization of macrophages through regulating miR-433-3p/GATA3 axis	(31)
GNAS-AS1	NSCLC	GNAS-AS1 promotes M2 polarization of macrophages via mediating miR-4319/NECAB3 signaling	(32)
MIR155HG	CRC	MIR155HG promote M2 polarization of macrophages by regulating the miR-650/ANXA2 axis	(33)
LINC00467	PC	LINC00467 promotes M2 polarization of macrophages by the miR-494-3p/STAT3 Axis	(34)
RP11-361F15.2	OS	RP11-361F15.2 promotes CPEB4-mediated tumorigenesis and M2-Like polarization of TAMs through binding with miR-30c-5p	(35)
NEAT1	MM	NEAT1 promotes M2 polarization of macrophage by sponging miR-214 and regulating B7-H3 via the JAK2/STAT3 signaling pathway	(36)
lINC00514	BC	Linc00514 promotes M2 polarization of TAMs via Jagged1-mediated notch signaling pathway through increasing phosphorylation of STAT3	(37)
CRNDE	Liver Cancer	CRNDE overexpression promotes M2 polarization of macrophages by activating JAK1/STAT6 signaling	(38)
MM2P	OC	MM2P knockdown blocks M2 polarization of macrophages by reducing phosphorylation of STAT6	(39)
SNHG1	BC	SNHG1 knockdown inhibits M2 polarization of macrophages by suppression of STAT6 phosphorylation	(40)
LINC00662	HCC	LINC00662 promotes M2 polarization of macrophages and HCC progression via activating Wnt/β-catenin signaling	(41)
XIST	Lung Cancer	XIST promotes M2 polarization Of macrophages through TCF-4	(42)
DCST1-AS1	OSCC	DCST1-AS1 promotes M2polarization of macrophages through activating NF-κB signaling	(43)
LincRNA-p21	BC	LincRNA-p21 knockdown facilitates M1polarization of macrophages by MDM2 inducing proteasome-dependent degradation of p53 and activating NF-κB and STAT3 pathway	(44)
LNMAT1	Bladder Cancer	LNMAT1 activates CCL2 expression by recruiting hnRNPL to CCL2 promoter to recruit macrophages into tumors	(45)
PTTG3P	CRC	HIF1A increases PTTG3P expression by binding to the PTTG3P promoter region to contribute to M2 phenotype of macrophage	(46)
LINC00665	GC	LINC00665 interacts with BACH1 to activate Wnt1 and mediates the M2 polarization of TAMs	(47)
PACERR	PDAC	PACERR induces pro-tumor macrophages via interacting with miR-671-3p and m6A-reader IGF2BP2	(48)
PACERR	PDAC	The CTCF/PACERR complex recruits E1A binding protein p300 to induce pro-tumor macrophages via directly regulating PTGS2 expression	(49)
ANCR	GC	ANCR overexpression inhibits M1 polarization by regulating FoxO1 expression	(50)
LINC01232	NSCLC	LINC01232 induced macrophages M2 polarization by activating TGF-β signaling pathway and recruiting IGF2BP2 to stabilize TGFBR1	(51)
NBR2	CRC	NBR2 overexpression alters M1/M2 phenotype transition by upregulating TNF-α and HLA-DR in M1 macrophages and suppressing Arg-1, CD163, and CD206 in M2 macrophages	(52)
NIFK-AS1	Endometrial Cancer	NIFK-AS1 inhibits the M2-like polarization of macrophages via targeting miR-146a	(53)
Lnc RNA cox-2	HCC	The lncRNA cox-2 siRNA decreases IL-12, iNOS, and TNF-α in M1 macrophages, increases IL-10, Arg-1, and Fizz-1 in M2 macrophages to alter M1/M2 macrophage polarization	(54)
CASC2c	GBM	CASC2c binds to FX and inhibits its expression and secretion, which in turn inhibits M2 polarization of macrophages	(55)
XIST	BC and OC	Silencing XIST alters M1/M2 macrophage polarization by miR-101/C/EBPα/KLF6 axis	(56)

TAMs, tumor-associated macrophages; CCA, Cholangiocarcinoma; ER+-BC, estrogen receptor positive Breast Cancer; NSCLC, Non-small Cell Lung Cancer; CRC, Colorectal Cancer; PC, Prostate Cancer; OS, Osteosarcoma; MM, Multiple Myeloma; BC, Breast Cancer; OC, Ovarian Cancer; HCC, Hepatocellular Carcinoma; OSCC, Oral Squamous Cell Carcinoma; GC, Gastric Cancer; PDAC, Pancreatic Ductal Adenocarcinoma; GBM, Glioblastoma multiforme.

### 2.1 LncRNAs function as oncogenes by modulating macrophage polarization and promoting tumor progression

LncRNAs are important contributors to the regulation of TME. The oncogenic roles of lncRNAs in TAMs and tumors involve different mechanisms, including sponging by the ceRNA network, classical signaling pathways, epigenetic modifications, and other regulatory pathways.

#### 2.1.1 The competing endogenous RNA network

The lncRNA-microRNA (miRNA)-mRNA network is a ceRNA network that is closely related to cancer initiation and progression in a variety of tumors, including glioma (58), breast cancer (BC) (59), lung cancer (60), gastric cancer (GC) (61), hepatocellular carcinoma (HCC) (62), colorectal cancer (CRC) (63), pancreatic cancer (64), cholangiocarcinoma (65), ovarian cancer (66), thyroid cancer (67), and clear cell renal cell carcinoma (68). LncRNAs can bind to miRNAs competitively and block miRNA interaction with the target mRNA, thereby indirectly elevating the expression and/or translation of target genes (69).

Several lncRNAs regulate TAM polarization and phenotypic transition *via* the ceRNA network. For example, in cholangiocarcinoma, the lncRNA prostate cancer-associated transcript 6 (PCAT6) mediates M2 polarization through a ceRNA network in which lncRNA PCAT6 suppresses the expression of miR-326 and promotes the expression of RhoA, a target gene of miR-326. Thus, PCAT6 could be a potential immunotherapy target for cholangiocarcinoma treatment (30).

Similarly, the lncRNA GNAS-AS1 accelerates M2 polarization by sponging miR-433-3p. GATA3, a transcription factor involved in M2 polarization, is a direct target of miR-433-3p.GNAS-AS1 positively regulates GATA3 through direct interaction and sponging of miR-433-3p, which results in TAM-mediated progression of estrogen receptor (ER)-positive BC. The effects of GNAS-AS1 on M2 polarization and the proliferation and metastasis of ER-positive BC cells can be inhibited through overexpression of miR-433-3p or GATA3 knockdown (31). In addition, increased expression of GNAS-AS1 in TAM inducesM2 polarization and accelerates non-small cell lung cancer (NSCLC) tumor progression (32).

In CRC, the lncRNA MIR155HG accelerates cancer progression and enhances oxaliplatin resistance by promoting M2 polarization as well as proliferation and metastasis of CRC cells by regulating the miR-650/ANXA2 axis (33). LINC00467 is an oncogenic lncRNA involved in M2 polarization and prostate carcinoma (PC) progression. LINC00467 is not only overexpressed in PC cells, but also in M2 macrophages, in which it enhances M2 polarization during PC progression. LINC00467 competitively binds to miR-494-3p and prevents the binding of miR-494-3p to STAT3, which functions as an oncogenic protein in many types of cancers. The repression of the LINC00467/miR-494-3p/STAT3 axis can halt the progression of PC by suppressing M2 polarization (34).

In osteosarcoma (OS), lncRNA RP11-361F15.2 is positively related to cytoplasmic polyadenylation element binding protein 4 (CPEB4) and negatively associated with miR-30c-5p. RP11-361F15.2 acts as an oncogenic factor that promotes CPEB4-mediated cancer cell migration and invasion by binding to miR-30c-5p and increasing CPEB4 protein levels. In addition, RP11-361F15.2 is required for the polarization of M2-like TAMs (35). In multiple myeloma, the tumorigenic lncRNA nuclear paraspeckle assembly transcript 1 (NEAT1) induces M2 polarization and promotes cancer progression by sponging miR-214 and upregulating the expression of B7-H3, a target of miR-214. The functional axis composed of NEAT1/miR-214/B7-H3 regulates M2 polarization and accelerates the progression of multiple myeloma (36).

#### 2.1.2 The classical signaling pathways

##### 2.1.2.1 Janus kinase/signal transducers and activators of transcription signaling pathway

In addition to the ceRNA network, lncRNAs also directly regulate signaling molecules associated with the classical pathways of M2 polarization, such as the JAK/STAT signaling pathway. This pathway has been widely studied in many cancers including BC (70), cervical cancer (71), oral and gastric cancer (72), HCC (73), pancreatic cancer (74), lung cancer (75), glioblastoma (76), melanoma (77), leukemia (78), lymphoma (79), and myeloproliferative neoplasms (80).

The JAK/STAT pathway participates in lncRNA-mediated activation of TAMs. In BC cells, the expression of Linc00514 is increased and it directly binds to STAT3 which is then recruited to JAK2, leading to an increased or sustained phosphorylation of STAT3 and activation of the Jagged1-mediatedNotch signaling pathway. Further, the STAT3/Jagged1 axis promotes the expression and secretion of IL-4 and IL-6 from BC cells, which induce M2 polarization in the TME (37). Therefore, JAK2/STAT3 is an important pathway in lncRNA-mediated M2 polarization.

JAK1/STAT6 is a key pathway of IL-4 and IL-13 induced M2 polarization, which can be regulated by lncRNAs. For example, high expression of lncRNA CRNDE activates JAK1 and STAT6 expression and upregulates the phospho-STAT6-dependent expression of CD163 and M2 polarization in liver cancer (38). In addition, lncRNA MM2P-mediated activation of STAT6 is required for the M2 polarization of macrophages and their angiogenesis-promoting properties. Mechanistically, lncRNA MM2P regulates the dephosphorylation of STAT6 (Y641) during M2 macrophage-mediated tumorigenesis (39). In contrast, the lncRNA SNHG1 contributes to M2 polarization and M2 macrophage-driven tumor growth and angiogenesis in BC by increasing the phosphorylation of STAT6 (40).

##### 2.1.2.2 Wnt/β-catenin signaling pathway

The Wnt/β-catenin pathway is one of the major oncogenic signaling cascades, and its dysregulation leads to cancer development, tumor growth, and dissemination (81, 82). Recent studies have shown that Wnt/β-catenin signaling is an important contributor to the lncRNA-mediated M2 polarization. The oncogenic lncRNA LINC00662 activates Wnt/β-catenin signaling by upregulating WNT3A expression *via* competitive binding with miR-15a, miR-16, and miR-107, which directly target and inhibit the expression and translation of WNT3A in HCC. Besides, the expression of LINC00662 in HCC activates Wnt/β-catenin signaling in macrophages in a paracrine manner by secreting WNT3A and promotingM2 polarization (41).

T-cell factor 4 (TCF-4) is a major effector and an important downstream transcriptional mediator of canonical Wnt signaling, which induces the expression of Wnt target genes. In lung cancer, TCF-4 positively regulates lncRNA XIST expression in TAM by directly binding to the promoter region of XIST gene. The upregulation of TCF-4/lncRNA XIST contributes to TAM phenotypic transformation and M2 polarization, which drive tumor progression in lung cancer (42).

##### 2.1.2.3 NF-κB signaling pathway

NF-κB signaling, a master regulator of immune responses that connects inflammation to cancer (83), is involved in the regulation of lncRNA-mediated M2 polarization. In oral squamous cell carcinoma (OSCC), the elevation of lncRNA DCST1-AS1 is required for the proliferation, migration, and invasion of OSCC, in addition to the upregulation of M2-like polarization. Loss of DCST1-AS1 inhibits OSCC tumorigenicity and represses M2 polarization through inactivation of NF-κB signaling. Thus, DCST1-AS1 modulates OSCC tumorigenicity and M2 polarization by activating the NF-κB pathway (43).

In BC, lincRNA-p21 is upregulated in TAMs and promotes M2 polarization. In the TME, lincRNA-p21 binds to MDM2 and blocks its interaction with p53, thereby increasing the stability and activity of p53, which in turn inhibits NF-κB and STAT3 pathways and maintains the M2 polarized state of macrophages. However, the silencing of lincRNA-p21 in TAM can inhibit the malignant properties and induce apoptosis in BC cells mainly through MDM2-dependent degradation of p53 and activation of the NF-κB/STAT3 pathways, which switch the polarization toM1 phenotype. Thus, the level of lincRNA-p21 is an important determinant of M1/M2 polarization in the TME during BC progression (44).

#### 2.1.3 The epigenetic modifications

Apart from post-transcriptional regulatory mechanisms discussed in previous sections, lncRNAs can act as scaffold molecules to modulate macrophage recruitment and polarization by controlling or guiding the transcription of target molecules. For instance, lncRNA lymph node metastasis-associated transcript 1 (LNMAT1) epigenetically induces C-Cmotif chemokine ligand (CCL) 2 expression by recruiting hnRNPL to the CCL2 promoter, which increases trimethylation of H3K4 at the promoter and enhances transcription of CCL2 in metastatic bladder cancer cells. Moreover, enhanced expression of CCL2 induces TAM activation and M2 polarization, which ultimately contribute to lymphangiogenesis and lymph node metastasis of bladder cancer cells by secreting vascular endothelial growth factor C (VEGF-C) (45).

The lncRNA pituitary tumor-transforming 3 pseudogene (PTTG3P) also promotes M2 polarization, which is correlated with hypoxia-inducible factor-1α (HIF-1α). HIF-1α can increase PTTG3P expression by binding to its promoter region and facilitating M2 polarization and cell growth (46). An oncogenic lncRNA,LINC00665, promotes M2 macrophage-dependent progression of GC by promoting the expression of Wnt1, which is a major signaling molecule involved in M2 polarization.LINC00665 directly interacts with the transcription factor BTB domain and CNC homology 1 (BACH1) and enhances the binding of BACH1 to the Wnt1 promoter, resulting in upregulation of Wnt1 expression (47).

LncRNA prostaglandin-endoperoxide synthase 2 (PTGS2) antisense NF-κB1 complex-mediated expression regulator RNA (PACERR) is an important contributor to the polarization of TAMs in pancreatic ductal adenocarcinoma (PDAC). PACERR acts as amiR-671-3p sponge to activate the KLF12/AKT/c-myc pathway and it also enhances the stability of KLF12 and c-myc by interacting with insulin-like growth factor 2 mRNA-binding protein 2 (IGF2BP2). KLF12 can directly bind to the promoter of PACERR, and KLF12-transcribed PACERR recruits histone acetyltransferase E1A binding protein p300 (EP300) to increase the acetylation of histone (H3K27) in the PACERR promoter region and enhance the transcription of PACERR in TAM (48). Notably, PTGS2 and PACERR have overlapping promoter regions, and PACERR is transcribed in the opposite direction of PTGS2; thus, PACERR functions as a *cis*-acting lncRNA in TAM. In addition, PACERR directly interacts with CCCTC-binding factor (CTCF), which is a DNA-binding factor involved in recruiting histone modifiers. CTCF/PACERR complexes recruit EP300 to the promoter region of PTGS2and increase histone acetylation, resulting in the enhancement of transcriptional activation of PTGS2 and induction of M2 polarization within the TME during the development and progression of PDAC (49).

Thus, some lncRNAs affect the activation and polarization of macrophages by functioning at the transcriptional level and/or assisting factors associated with epigenetic modifications.

#### 2.1.4 Other regulatory mechanisms

LncRNAs modulate the polarization state of macrophages *via* other mechanisms. For example, upregulation of oncogenic lncRNA ANCR in GC accelerates the invasion and metastasis by inhibiting macrophage M1 polarization by triggering ubiquitination-mediated degradation and downregulation of FOX protein O1 (FoxO1) (50).

In NSCLC, increased LINC01232 expression is closely associated with the stemness of NSCLC cells and cancer progression through the activation of the transforming growth factor-beta (TGF-β) signaling pathway. Forkhead box P3 (FOXP3) acts as an upstream activator of LINC01232 transcription, and FOXP3-dependent expression of LINC01232 accelerates M2 polarization by LINC01232/IGF2BP2 mediated increase in TGFBR1 mRNA stability and elevation of TGFBR1 protein levels (51).

LncRNAs thus function as oncogenes by modulating the polarization of macrophages *via* multiple mechanisms, thus accelerating the occurrence and progression of tumors.

### 2.2 LncRNAs function as tumor suppressors by modulating macrophage polarization and inhibiting tumor progression

LncRNAs act as tumor suppressors by altering the macrophage M1/M2 phenotypic switch, mainly by inducing M1 polarization and inhibiting M2 macrophage expression, thus inhibiting cancer cell proliferation, invasion, migration, and immune evasion. For instance, the lncRNA neighbor of BRCA1 gene 2 (NBR2) is downregulated during the development of CRC and exerts an antitumor effect by favoring M1 polarization and suppressing M2 polarization of TAM. The overexpression of lncRNA NBR2 increased the proportion of M1 macrophages and upregulated the expression levels of TNF-α and HLA-DR in the TME of the CRC xenograft model, indicating that lncRNA can suppress CRC progression by altering the M1/M2 polarization (52). Similarly, the reduction of lncRNA NIFK-AS1 in TAM of endometrial cancer leads to miR-146a-mediated suppression of Notch1 signaling, which promotes M2-like macrophage-driven proliferation, migration, and invasion of cancer cells. However, the overexpression of lncRNA NIFK-AS1 can suppress cancer growth by sponging miR-146a and upregulating Notch1, thus inhibiting estrogen-induced proliferation, migration, and invasion of endometrial cancer cells (53).In addition, the expression of lncRNA cox-2 is higher in M1 type than in M2 type macrophages. The expression of lncRNA cox-2 in M1 type macrophages upregulates the levels of TNF-α, IL-12 and iNOS, which facilitates HCC cell apoptosis and increases the capability of M1 macrophages to inhibit growth, migration, and invasion of HCC cells. Therefore, lncRNA cox-2 can suppress HCC immune evasion and tumor growth by blocking the M2 polarization (54).

Coagulation factor X (FX), a vitamin K-dependent plasma protein, is overexpressed in glioblastoma multiforme (GBM).Secreted FX, a chemoattractant, is closely associated with TAM density and specifically increases the M2 macrophages in GBM.FX facilitates macrophage recruitment and M2 polarization to accelerate GBM growth without altering proliferation by increasing the phosphorylation and activation of ERK1/2 and AKT in TAM. The tumor-suppressive miR-338-3p targets FX and suppresses macrophage migration without affecting their polarization. However, lncRNA CASC2c directly inhibits FX expression and blocks macrophage migration and M2 polarization. Interestingly, miR-388-3p and lncRNA CASC2c reciprocally regulate each other and synergistically repress FX expression, which leads to the reduction of the M2 subtype and elevation of antitumor responsive M1 subtype macrophages in GBM (55).

LncRNA XIST is highly expressed and contributes to maintaining the M1 phenotype by increasing the expression of CCAAT/enhancer-binding protein α (C/EBPα) and Kruppel-like factor 6 (KLF6), which are important regulators and inhibitors of the formation of M2 macrophages, while miR-101, an oncogenic miR, can inhibit the expression of C/EBPα and KLF6 mRNAs and promote M2 polarization. However, the expression of lncRNA XIST is lowered and miR-101 is upregulated in TAMs of breast and ovarian cancers, favoring M2 polarization-induced cell proliferation and migration of malignant cells. Notably, C/EBPα and KLF6 have a common miRNA response element (MRE) sequence for the binding of XIST and miR-101, and miR-101 can also directly bind to XIST. Hence, XIST competes with miR-101 to bind with C/EBPα and KLF6 and sponges miR-101 to upregulate the expression of C/EBPα and KLF6 in TAM of ovarian cancer and BC. Therefore, tumor-suppressive lncRNAs competitively block miRNA-mediated M2 polarization and control the M1/M2 phenotypic transformation of TAMs (56).

## 3 TAMs regulate LncRNAs to affect tumorigenesis and progression

**Figure 2:**
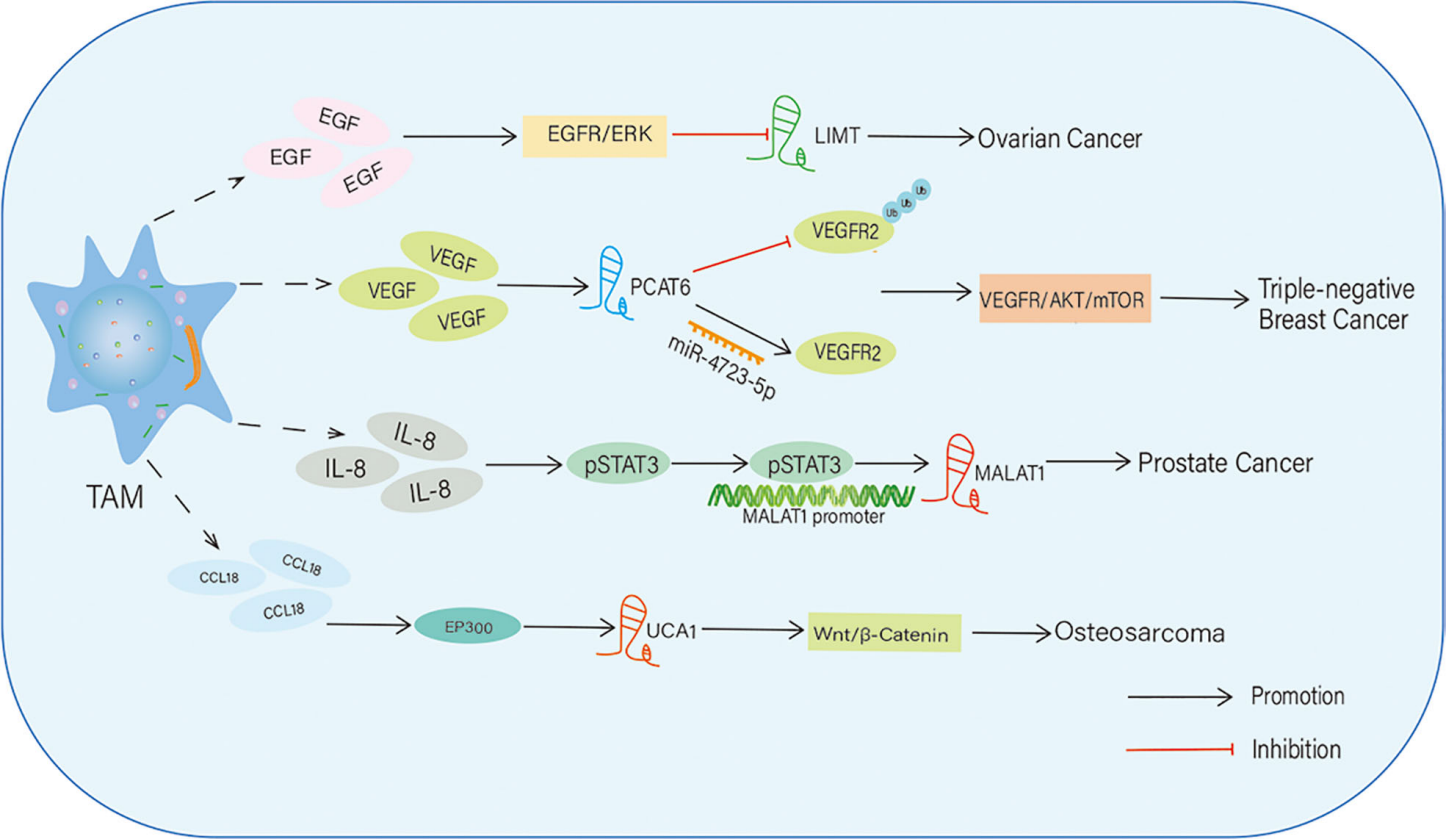
TAMs regulate the expression level of IncRNAs in tumors. TAMs upregulate or downregulate the expression of IncRNAs by secreting multiple cytokines. Then, these IncRNAs affect tumor progression through activating various signaling pathways.

In some tumors, TAMs mediate the expression of lncRNAs by releasing cytokines and eventually influence the onset and development of tumors (**Table 1**) (18, 29). M2-like TAMs are recruited to tumor tissues and secrete growth factors, cytokines, chemokines, and anti-inflammatory mediators (84). These factors can alter the expression levels of lncRNAs, thereby influencing growth, invasion, metastasis, and angiogenesis. Therefore, in this section, we summarize and discuss findings related to TAM-mediated regulation of lncRNAs in tumors (**Figure 2**).

In epithelial ovarian cancer (EOC), a stimulant, phorbol 12-myristate 13-acetate, induces M2-like TAMs and triggers the secretion of epidermal growth factor (EGF), which promotes proliferation, migration, invasion, and epithelial-mesenchymal transition (EMT) of EOC cells *via* activation of the EGFR-ERK signaling pathway. In EOC, EGF-induced EGFR-ERK signaling inhibits the expression of the lncRNA inhibiting metastasis (LIMT), a highly conserved tumor suppressive lncRNA. Forced expression of LIMT or inhibition of EGFR signaling significantly reduced ovarian tumor growth. Thus, the secretion of growth factors such as EGF from M2-like TAMs induces EGFR signaling-mediated suppression of lncRNA expression in EOC and accelerates EOC progression and metastasis (85).

VEGF is a vital angiogenesis factor that stimulates endothelial cell proliferation and tube formation to generate new blood vessels; thus, it is an important contributor to tumor angiogenesis (86). In triple-negative BC (TNBC), VEGF secreted from M2 macrophages stimulates the expression of the lncRNA PCAT6 in TNBC cells. PCAT6 post-transcriptionally upregulates the level of VEGFR2 by targeting and sponging miR-4723-5p, and the VEGFR2/AKT/mTOR signaling pathway acts as a downstream effector of PCAT6 to accelerate tumorigenesis and angiogenesis. In addition, PCAT6 sustains VEGFR2 signaling by recruiting a deubiquitinase enzyme (USP14) and inhibiting ubiquitination-dependent degradation of VEGFR2 protein. Together, this study reveals that M2 macrophages secrete growth factors such as VEGF, which have paracrine effects on BC cells, upregulate oncogenic signaling composed of PCAT6/VEGFR2/AKT/mTOR, and trigger tumor progression (87).

TAMs also release various chemokines to modulate lncRNA expression in some tumors. For instance, IL-8, a CXC inflammatory chemokine, is secreted by M2 macrophages, which induces angiogenesis and tumor progression in prostate cancer by STAT3-dependent expression of lncRNA metastasis-associated with lung adenocarcinoma transcript-1 (MALAT1), which is an oncogenic lncRNA. STAT3 enhances MALAT1 transcription by directly binding to its promoter. This study identified that the IL-8/STAT3/MALAT1 regulatory axis is activated by M2 macrophages in a paracrine manner (88). CCL 18 is a chemokine secreted by TAMs, and its expression is associated with metastasis in many types of tumors (89, 90). TAM-secreted CCL18 promotes the proliferation and migration of OS cells *via* EP300 and mediates the expression of the lncRNA UCA1.In addition, the Wnt/β-catenin/GSK3β signaling pathway is a downstream effector of lncRNA UCA1 during OS progression and metastasis. In this way, TAM-derived CCL18 facilitates OS proliferation and metastasis through EP300-mediated expression of UCA1 and activation of the Wnt/β-catenin axis (91).

Collectively, these studies reveal that some lncRNAs affect the expression and phenotype transition of TAMs, while TAMs can influence lncRNA expression in other cells within the TME to influence tumor progression; in some cases, there is a reciprocal relationship between TAM and lncRNAs, and their interaction forms a positive/negative feedback loop, functioning as oncogenes/tumor suppressors in cancers. For example, TAMs facilitate OS progression by enhancing the expression of lncRNA p53 upregulated regulator of P53 (PURPL) by influencing the miR-363/PDZ domain containing 2 (PDZD2) axis. PURPL positively regulates TAM migration and promotes OS cell proliferation, migration, invasion, and EMT (92). This supports the notion that there is feedback regulation and/or crosstalk between lncRNAs and TAMs in tumors.

## 4 The involvement of sEV in the crosstalk between LncRNAs and TAMs

LncRNAs are important participants in the crosstalk between tumor cells and macrophages, and they can be transferred along with multiple cellular components through intercellular messengers, such as sEV and microvesicles (MVs) (57). The sEV are 40-160 nm in diameter and are secreted by all cell types. They are vital for information exchange between cells in normal tissues as well as in the TME (8, 93). They carry multiple biological molecules including lncRNAs and play crucial roles in tumor progression (94–102). Tumor-derived lncRNAs, such as PTENP1 (103), LNMAT2 (104), lncUEGC1 (105), and FMR1-AS1 (106), can be delivered by sEV, which influences the onset and development of tumors. Some sEV carrying lncRNAs can be used as tumor biomarkers and potential therapeutic targets. In this section, we focus on the interactions among sEV, lncRNAs, and TAMs during tumorigenesis and dissemination.

### 4.1 The roles of tumor-derived sEV in the crosstalk between lncRNAs and TAMs

Cancer cell-derived sEV can transport lncRNAs into macrophages to mediate M2 polarization within the TME (107). Subsequently, polarized TAMs can trigger cancer cell growth and tumor progression by upregulating the levels of anti-inflammatory cytokines and/or decreasing pro-inflammatory cytokines (**Table 2**). For instance, the oncogenic lncRNA HLA complex group 18 (HCG18) (115, 116), is highly expressed in GC cell-derived sEV. By transferring HCG18 through sEV, GC cells facilitate the M2 polarization by sponging miR-875-3p and upregulating KLF4 expression (108). The lncRNA TP73-AS1, released from nasopharyngeal carcinoma (NPC) cells, is transferred by sEV to macrophages, in which TP73-AS1 increases the levels of M2 markers and promotes their motility and tube formation. In addition, overexpression of TP73-AS1 accelerated NPC cell proliferation, colony formation, and DNA synthesis by directly binding to miR-342-3p. Thus, the NPC cell-derived sEV carrying lncRNATP73-AS1 accelerate oncogenic processes by promoting M2 polarization (109).

**Table 2:** The regulatory effect of lncRNAs in sEV derived from tumor cells on TAMs.

The source of sEV(tumor types)	Related lncRNA	The effects on TAMs and possible mechanism	reference
GC	HCG18	sEV-transmitted HCG18 facilitates M2 polarization of macrophages through elevating KLF4 expression by inhibiting miR-875-3p	(108)
NPC	TP73-AS1	sEV-transmitted TP73-AS1promotes M2 polarization of macrophages through targeting miR-342-3p	(109)
NSCLC	SOX2-OT	sEV-transmitted SOX2-OT promotes M2 polarization of macrophages via inducing Smads expression through sponging miR-627-3p	(110)
HCC	DLX6-AS1	HCC-derived sEV transported DLX6-AS1 into macrophages to induce M2 polarization of macrophages through regulating miR-15a-5p/CXCL17 axis	(111)
HCC	TUC339	sEV-transmitted TUC339 alters M1/M2 macrophages polarization through reducing M1 marker expression and enhancing M2 markers expression	(112)
CRC	RPPH1	sEV-transmitted RPPH1 promotes M2 polarization of macrophages through upregulating M2 markers	(113)
HNSCC	HOTTIP	HOTTIP induces macrophages toward the M1 phenotype via upregulating the TLR5/NF-κB signaling pathway through sponging miR-19a-3p and miR-19b-3p	(114)

sEV, Small Extracellular Vesicles; TAMs, Tumor-associated macrophages; GC, Gastric Cancer; NPC, Nasopharyngeal Carcinoma; NSCLC, Non-small Cell Lung Cancer;HCC, Hepatocellular Carcinoma;CRC, Colorectal Cancer; HNSCC, Head and Neck Squamous cell Carcinoma.

In NSCLC, the level of the lncRNA SOX2 overlapping transcript (SOX2-OT) is high in tumor-derived sEV, which play a crucial role in cancer development and progression. On the one hand, the lncRNA SOX2-OT, transmitted by NSCLC cell-derived sEV, promotes M2 polarization by sponging miR-627-3p and increasing the expression of Smad signaling molecules such as Smad2, Smad3, and Smad4 in macrophages. On the other hand, SOX2-OT-induced M2 macrophages aggravate the resistance of NSCLC cancer cells to EGFR tyrosine kinase inhibitors. This study revealed a close connection among tumor cell-derived lncRNAs, sEV, and M2 macrophages in NSCLC (110).

The lncRNA distal-less homeobox 6 antisense 1 (DLX6-AS1) is overexpressed in HCC and can trigger the migration, invasion, and EMT of HCC. HCC cell-secreted sEV transport DLX6-AS1 to macrophages and induce M2 polarization by regulating the miR-15a-5p/CXCL17 axis (111). Likewise, lncRNA TUC339 is primarily derived from HCC cell-derived sEV and can control M1/M2 polarization. In THP-1 cells, suppression of TUC339 elicits M1 phenotypic behavior of macrophages, as evidenced by increased levels of pro-inflammatory cytokines, costimulatory molecules, and phagocytosis. However, overexpression of TUC339 leads to the opposite effect, and macrophages acquire the M2 phenotype. Moreover, TUC339 levels are elevated in M2 macrophages and downregulated during the transition from M2 to M1 phenotype (112). Overall, this study indicated that HCC-derived sEV carrying lncRNA TUC339 participate in the M1/M2 transition of macrophages and M2 polarization in HCC.

Similarly, the lncRNA RPPH1 is abundant in circulatory sEV in CRC patients. TheRPPH1 carrying sEV, secreted by CRC cells, can be rapidly taken up by human monocyte-derived macrophages, and they exhibit a CD206 ^high/^HLA-DR ^low^ phenotype, a typical M2 macrophage morphology, and express M2 markers. This study suggests that CRC-derived sEV can transmit RPPH1 into macrophages to promote the M2 polarization, in addition to their function in CRC cell metastasis (113).

Together, cancer cell-secreted sEVs can transport lncRNAs to TAMs within the TME and promote M2 polarization, which triggers tumor progression. However, some studies have found that sEV carrying lncRNAs in the TME have the potential to induce macrophages towards the M1 phenotype, which functions as a suppressor of tumor progression. For example, the lncRNA HOXA transcript at the distal tip (HOTTIP) is upregulated in M1 macrophage-derived sEV (M1 sEV) in head and neck squamous cell carcinoma (HNSCC), and these M1 sEV inhibit proliferation, migration, and invasion, as well as promote apoptosis of HNSCC cells. This effect is enhanced by HOTTIP overexpression in M1 sEV, indicating that HOTTIP is an important functional molecule in M1 sEV. Notably, M1 sEV could reprogram TAMs in the TME into M1 macrophages, and HOTTIP-carrying sEV from both cancer cells and M1 macrophages could reprogram circulating monocytes to express the M1 phenotype. M1 sEV carrying HOTTIP also upregulates the TLR5/NF-κB signaling pathway by competitively sponging miR-19a-3p and miR-19b-3p to suppress HNSCC progression (114).

### 4.2 The roles of macrophages-derived sEV in the crosstalk between lncRNAs and TAMs

**Figure 3:**
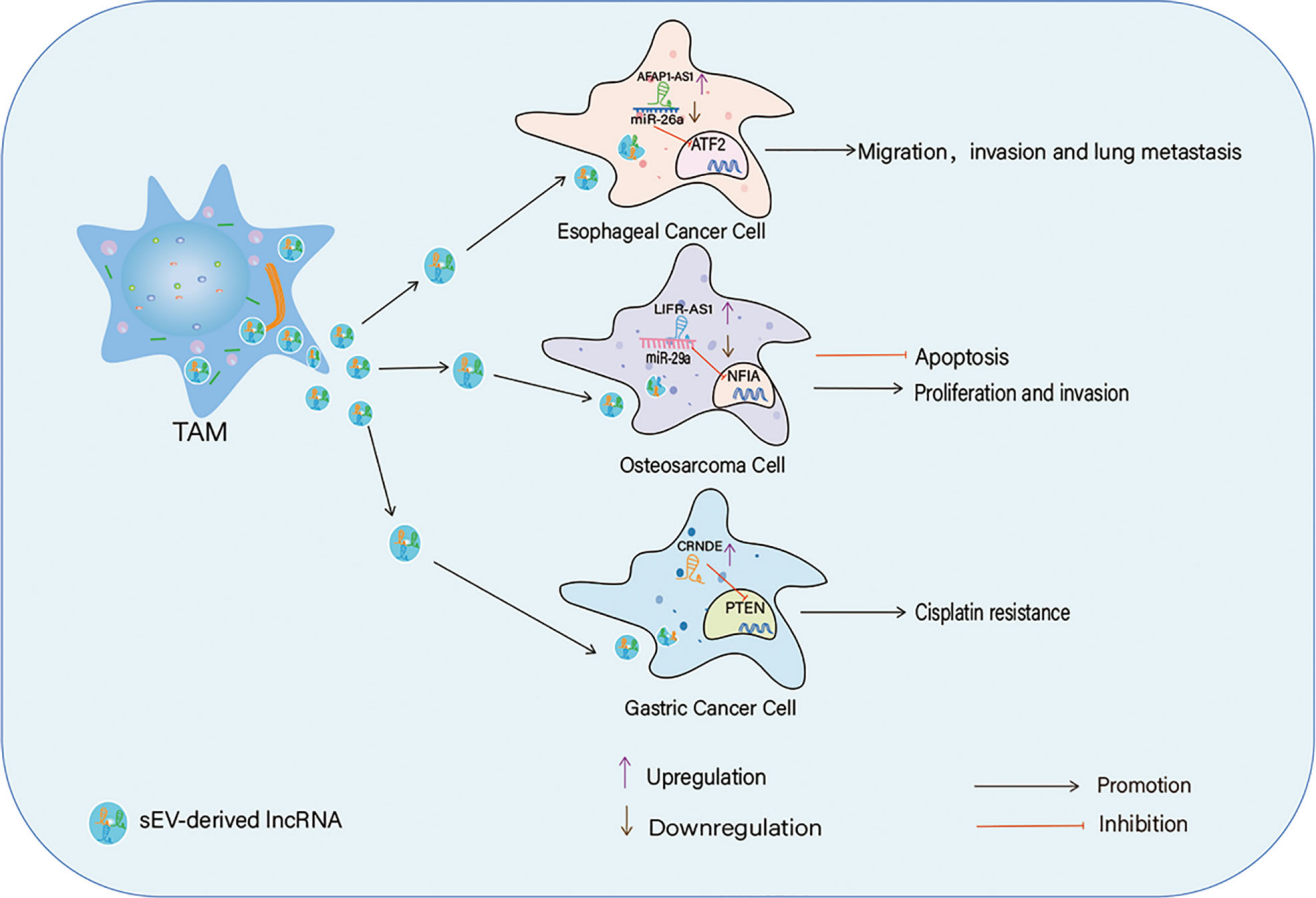
TAMs-derived sEV in the crosstalk between IncRNAs and TAMs, TAMs secret sEV to transport IncRNAs into cancer cells to affect cancer cells proliferation, apotosis, migration, invasion and drug resistance through different ways.

In the TME, sEV are released not only by tumor cells but also by immune cells such as TAMs. TAMs can secrete and utilize sEV to modulate the expression of lncRNAs, thus altering the signaling associated with cancer initiation and progression **Figure 3**. In esophageal cancer (EC), M2 macrophage-derived sEV consist of high levels of lncRNA AFAP1-AS1 and activating transcription factor 2 (ATF2) but a decreased level of miR-26a. AFAP1-AS1 can be packed into EC cells by these sEV and upregulates the expression of ATF2 by sponging miR-26a, thereby promoting the migration, invasion, and lung metastasis of EC cells (117). Similarly, macrophage-derived lncRNA LIFR-AS1 can be transferred to OS cells *via* sEV to promote OS cell proliferation and invasion through the miR-29a/nuclear factor I/A (NFIA) axis (118). The level of lncRNA CRNDE is high in cancer tissues and TAMs of GC patients. A study found that CRNDE is enriched in M2 macrophage-derived sEV, and its transportation to GC cells *via* sEV enhances cisplatin resistance of GC cells by activating neural precursor cells expressing developmentally downregulated protein 4-1 (NEDD4-1)-mediated ubiquitination and degradation of phosphatase and tensin homolog (PTEN). Thus, TAM-derived sEV carrying lncRNAs, such as CRNDE, participate in chemoresistance in GC (119).

In some tumors, TAM-secreted sEV can suppress cancer cell migration and invasion. The sEV from TAM of EOC carrying miR-146b-5p when taken up by human umbilical vein derived endothelial cells effectively suppressed the migration of these endothelial cells by targeting the TRAF6/NF-κB/MMP2 pathway. However, two uncharacterized lncRNAs (ENST00000444164 and ENST00000437683) from EOC-derived sEV reversed endothelial cell migration by upregulating the phosphorylation of NF-κB. This study suggests that there is an interaction between TAMs and endothelial cells, and that certain TAM-derived miRs, such as miR-146b-5p, have anti-angiogenic functions by which endothelial cell migration is suppressed. However, EOC cells abrogate this hindrance through the secretion of sEV carrying oncogenic/angiogenic lncRNAs and promote endothelial cell migration, which is a key event in tumor angiogenesis (120).

Interactions among sEV, lncRNAs, and TAMs have also been found in lung adenocarcinoma (LUAD). LUAD cell-derived sEV deliver miR-19b-3p into macrophages, which targets protein tyrosine phosphatase receptor type D (PTPRD) and inhibits PTPRD-dependent dephosphorylation of STAT3. Sustained activation ofSTAT3 triggers M2 polarization in addition to LINC00273 transcription. Subsequently, the M2 macrophages secrete and transfer sEV carrying LINC00273 to LUAD cells, in which LINC00273 induces ubiquitination and degradation of large tumor suppressor kinase 2 (LATS2) by recruiting E3 ubiquitin-protein ligase (NEDD4) and activates YAP. Activated YAP promotes the transcription of RBMX, which binds to miR-19b-3p and facilitates its packaging into sEV from LUAD cells (121). Thus, TAMs and LUAD cells cooperatively function in the TME by this novel mechanism, and sEV are vital for the interactive communication between them.

## 5 Conclusions and perspectives

LncRNAs, sEV, and TAMs have been extensively studied in multiple tumors. Abnormal expression of lncRNAs is found in various body fluids, including blood, saliva, urine, and breast milk; hence, they can be used as biomarkers to predict cancer occurrence and progression. LncRNAs function as oncogenes or tumor suppressors and regulate cancer cell proliferation, apoptosis, invasion, metastasis, and drug resistance (3). Thus, a specific treatment strategy for silencing oncogenic lncRNAs or overexpressing tumor-suppressive lncRNAs should be developed to utilize potential lncRNA-based therapeutics in tumors. Several effective and efficient strategies, including siRNAs, CRISPR-Cas9, and antisense oligonucleotides (ASOs), have been used to suppress oncogenic lncRNAs. Among these strategies, ASOs regulate both target RNA processing and protein expression through different mechanisms, making this the most effective and promising strategy (122). ASO can be used to inhibit the expression of lncRNAs, thus preventing carcinogenic progression. For instance, the progression of HNSCC can be effectively blocked by silencing AC104041.1, using ASOs (123). Likewise, ASO-targeting lncRNA NRAD1 effectively inhibits tumor growth and suppresses the ability of tumor cells to acquire and maintain stem cell characteristics in TNBC (124). In addition, specific ASOs targeting lncRNA TROJAN can reduce lung metastatic nodules in TNBC (125).

LncRNAs can affect tumor development and progression by interacting with different cells in the TME (126, 127). TAMs occupy an important place in the TME, and there are close interactions between lncRNAs and TAMs that modulate tumorigenesis and metastasis. There is a reciprocal relationship between lncRNAs and the oncogenic functions of TAM. Some lncRNAs can influence the recruitment, polarization, and phenotypic transition of TAMs, whereas TAMs can alter the expression of lncRNAs by secreting cytokines and/or growth factors. Therefore, targeting TAMs is an effective strategy for tumor treatment. Based on the characteristics of TAMs, possible therapeutic strategies may include inhibiting the recruitment and activation of monocytes, reprogramming TAMs into an antitumor phenotype (M1), and/or targeting TAM-specific markers (18).

Intercellular information exchange occurs *via* sEV and they are key messengers in regulating the TME and contributing to tumor growth, invasion, metastasis, and drug resistance (128). The cargos of sEV, in particular, the nucleic acid content, are mostly similar to their parent cells (129). Furthermore, sEV are stable, have a long half-life in circulation, and protect their cargo from degradation by proteases and nucleases, which make them ideal biological components in liquid biopsies. Therefore, sEV and their cargo are considered better and valuable diagnostic and prognostic markers for various tumors, including lung cancer (130), BC (131), GC (132), HCC (133), CRC (134), pancreatic cancer (135), prostate cancer (136), EOC (137), head and neck cancer (138), and glioma (139). Therefore, it is possible to halt and/or block tumor progression by targeting and modulating sEV expression. This strategy may provide new ideas and offer new treatment options for tumor immunotherapy.

Although sEV are secreted by different types of cells, tumor-secreted sEV play a crucial role in macrophage polarization and cancer growth progression. For example, sEV secreted by lung cancer cells induce the transformation of macrophages into an M2 phenotype, which triggers immune suppression in the TME and tumor growth (140). In contrast, BC cell-derived sEV contribute to pre-metastatic niche formation and facilitate the bone metastasis of tumor cells (141). In addition, the contents of tumor-derived sEV, particularly lncRNAs, are involved in the interaction between tumor cells and TAMs. Additionally, TAMs can secrete and utilize sEV to transfer lncRNAs into tumor cells to exert their effects on tumor growth. In conclusion, sEV, lncRNAs, and TAMs can interact with each other and that these interactions have a direct impact on tumor occurrence and progression. Therefore, an in-depth understanding of the close relationship among these three factors in the TME not only provides potential and efficient therapeutic targets for tumor immunotherapy but also helps in the design of cancer-diagnostic and cancer-prognostic tools.

## Author contributions

L-JZ and FC designed the literature and originally wrote the manuscript. X-RL, MP and HQ edited and embellished the manuscript. Z-JL approved the final version of the manuscript. All authors read and approved the submitted version.

## Funding

The study was funded by the National Natural Science Foundation of China (32000495), National Natural Science Foundation of Shandong Province (ZR2020MH202).

## Conflict of interest

The authors declare that the research was conducted in the absence of any commercial or financial relationships that could be construed as a potential conflict of interest.

## Publisher’s note

All claims expressed in this article are solely those of the authors and do not necessarily represent those of their affiliated organizations, or those of the publisher, the editors and the reviewers. Any product that may be evaluated in this article, or claim that may be made by its manufacturer, is not guaranteed or endorsed by the publisher.

## References

[1] Kaikkonen MU Adelman K. Emerging roles of non-coding RNA transcription. Trends Biochem Sci (2018) 43:654–67. doi: 10.1016/j.tibs.2018.06.002 30145998

[2] Anastasiadou E Jacob LS Slack FJ. Non-coding RNA networks in cancer. Nat Rev Cancer (2018) 18:5–18. doi: 10.1038/nrc.2017.99 29170536 PMC6337726

[3] Slack FJ Chinnaiyan AM. The role of non-coding RNAs in oncology. Cell (2019) 179:1033–55. doi: 10.1016/j.cell.2019.10.017 PMC7347159 31730848

[4] Liu Y Liu X Lin C Jia X Zhu H Song J. Noncoding RNAs regulate alternative splicing in cancer. J Exp Clin Cancer Res (2021) 40:11. doi: 10.1186/s13046-020-01798-2 33407694 PMC7789004

[5] Wong CM Tsang FH Ng IO. Non-coding RNAs in hepatocellular carcinoma: Molecular functions and pathological implications. Nat Rev Gastroenterol Hepatol (2018) 15:137–51. doi: 10.1038/nrgastro.2017.169 29317776

[6] Mohapatra S Pioppini C Ozpolat B Calin GA. Non-coding RNAs regulation of macrophage polarization in cancer. Mol Cancer (2021) 20:24. doi: 10.1186/s12943-021-01313-x 33522932 PMC7849140

[7] Sanchez Calle A Kawamura Y Yamamoto Y Takeshita F Ochiya T. Emerging roles of long non-coding RNA in cancer. Cancer Sci (2018) 109:2093–100. doi: 10.1111/cas.13642 PMC6029823 29774630

[8] Sun Z Yang S Zhou Q Wang G Song J Li Z. Emerging role of exosome-derived long non-coding RNAs in tumor microenvironment. Mol Cancer (2018) 17:82. doi: 10.1186/s12943-018-0831-z 29678180 PMC5909226

[9] Ping L Zhang K Ou X Qiu X Xiao X. A novel pyroptosis-associated long non-coding RNA signature predicts prognosis and tumor immune microenvironment of patients with breast cancer. Front Cell Dev Biol (2021) 9:727183. doi: 10.3389/fcell.2021.727183 34616734 PMC8488148

[10] Zhang H Zhao L Li S Wang J Feng C Li T. N6-Methylandenosine-Related lncRNAs in tumor microenvironment are potential prognostic biomarkers in colon cancer. Front Oncol (2021) 11:697949. doi: 10.3389/fonc.2021.697949 34178697 PMC8231021

[11] Hinshaw DC Shevde LA. The tumor microenvironment innately modulates cancer progression. Cancer Res (2019) 79:4557–66. doi: 10.1158/0008-5472.CAN-18-3962 PMC6744958 31350295

[12] Vitale I Manic G Coussens LM Kroemer G Galluzzi L. Macrophages and metabolism in the tumor microenvironment. Cell Metab (2019) 30:36–50. doi: 10.1016/j.cmet.2019.06.001 31269428

[13] Han D Fang Y Guo Y Hong W Tu J Wei W. The emerging role of long non-coding RNAs in tumor-associated macrophages. J Cancer (2019) 10:3389/fonc.2021.810893. doi: 10.7150/jca.3577010.3389/fonc.2021.810893 31777603 PMC6856883

[14] Xu J Liu XY Zhang Q Liu H Zhang P Tian ZB. Crosstalk among YAP, LncRNA, and tumor-associated macrophages in tumorigenesis development. Front Oncol (2021) 11:810893. doi: 10.3389/fonc.2021.810893 35071016 PMC8770286

[15] Na YR Kwon JW Kim DY Chung H Song J Jung D. Protein kinase a catalytic subunit is a molecular switch that promotes the pro-tumoral function of macrophages. Cell Rep (2020) 31:107643. doi: 10.1016/j.celrep.2020.107643 32402274

[16] Giurisato E Xu Q Lonardi S Telfer B Russo I Pearson A. Myeloid ERK5 deficiency suppresses tumor growth by blocking protumor macrophage polarization *via* STAT3 inhibition. Proc Natl Acad Sci USA (2018) 115:E2801–10. doi: 10.1073/pnas.1707929115 PMC5866536 29507229

[17] Wu L Tang H Zheng H Liu X Liu Y Tao J. Multiwalled carbon nanotubes prevent tumor metastasis through switching M2-polarized macrophages to m1 *via* TLR4 activation. J BioMed Nanotechnol (2019) 15:138–50. doi: 10.1166/jbn.2019.2661 30480521

[18] Pan Y Yu Y Wang X Zhang T. Tumor-associated macrophages in tumor immunity. Front Immunol (2020) 11:583084. doi: 10.3389/fimmu.2020.583084 33365025 PMC7751482

[19] Li C Xu X Wei S Jiang P Xue L Wang J. Tumor-associated macrophages: Potential therapeutic strategies and future prospects in cancer. J Immunother Cancer (2021) 9:e1341. doi: 10.1136/jitc-2020-001341 PMC8728363 33504575

[20] Lin Y Xu J Lan H. Tumor-associated macrophages in tumor metastasis: Biological roles and clinical therapeutic applications. J Hematol Oncol (2019) 12:76. doi: 10.1186/s13045-019-0760-3 31300030 PMC6626377

[21] Jeong H Kim S Hong BJ Lee CJ Kim YE Bok S. Tumor-associated macrophages enhance tumor hypoxia and aerobic glycolysis. Cancer Res (2019) 79:795–806. doi: 10.1158/0008-5472.CAN-18-2545 30610087

[22] Maas SLN Breakefield XO Weaver AM. Extracellular vesicles: Unique intercellular delivery vehicles. Trends Cell Biol (2017) 27:172–88. doi: 10.1016/j.tcb.2016.11.003 PMC5318253 27979573

[23] Thery C Witwer KW Aikawa E Alcaraz MJ Anderson JD Andriantsitohaina R. Minimal information for studies of extracellular vesicles 2018 (MISEV2018): A position statement of the international society for extracellular vesicles and update of the MISEV2014 guidelines. J Extracell Vesicles (2018) 7:1535750. doi: 10.1080/20013078.2018.1535750 30637094 PMC6322352

[24] Wang H You Y Zhu X. The role of exosomes in the progression and therapeutic resistance of hematological malignancies. Front Oncol (2022) 12:887518. doi: 10.3389/fonc.2022.887518 35692747 PMC9178091

[25] Hoshino A Costa-Silva B Shen TL Rodrigues G Hashimoto A Tesic MM. Tumour exosome integrins determine organotropic metastasis. Nature (2015) 527:329–35. doi: 10.1038/nature15756 PMC4788391 26524530

[26] Das GA Krawczynska N Nelson ER. Extracellular vesicles-the next frontier in endocrinology. Endocrinology (2021) 162 :bqab133. doi: 10.1210/endocr/bqab133 34180968 PMC8294678

[27] Li I Nabet BY. Exosomes in the tumor microenvironment as mediators of cancer therapy resistance. Mol Cancer (2019) 18 :32. doi: 10.1186/s12943-019-0975-5 30823926 PMC6397467

[28] Mashouri L Yousefi H Aref AR Ahadi AM Molaei F Alahari SK. Exosomes: Composition, biogenesis, and mechanisms in cancer metastasis and drug resistance. Mol Cancer (2019) 18:75. doi: 10.1186/s12943-019-0991-5 30940145 PMC6444571

[29] Wu K Lin K Li X Yuan X Xu P Ni P. Redefining tumor-associated macrophage subpopulations and functions in the tumor microenvironment. Front Immunol (2020) 11:1731. doi: 10.3389/fimmu.2020.01731 32849616 PMC7417513

[30] Tu J Wu F Chen L Zheng L Yang Y Ying X. Long non-coding RNA PCAT6 induces m2 polarization of macrophages in cholangiocarcinoma *via* modulating miR-326 and RhoA-ROCK signaling pathway. Front Oncol (2021) 10:605877. doi: 10.3389/fonc.2020.605877 33552977 PMC7859434

[31] Liu SQ Zhou ZY Dong X Guo L Zhang KJ. LncRNA GNAS-AS1 facilitates ER+ breast cancer cells progression by promoting M2 macrophage polarization *via* regulating miR-433-3p/GATA3 axis. Biosci Rep (2020) 40 :BSR20200626. doi: 10.1042/BSR20200626 32538432 PMC7327181

[32] Li Z Feng C Guo J Hu X Xie D. GNAS-AS1/miR-4319/NECAB3 axis promotes migration and invasion of non-small cell lung cancer cells by altering macrophage polarization. Funct Integr Genomic (2020) 20:17–28. doi: 10.1007/s10142-019-00696-x 31267263

[33] Zhou L Li J Liao M Zhang Q Yang M. LncRNA MIR155HG induces M2 macrophage polarization and drug resistance of colorectal cancer cells by regulating ANXA2. Cancer Immunology Immunother (2021) 71 :1075-91. doi: 10.1007/s00262-021-03055-7 PMC10991596 34562123

[34] Jiang H Deng W Zhu K Zeng Z Hu B Zhou Z. LINC00467 promotes prostate cancer progression *via* m2 macrophage polarization and the miR-494-3p/STAT3 axis. Front Oncol (2021) 11:661431. doi: 10.3389/fonc.2021.661431 34094954 PMC8170392

[35] Yang D Liu K Fan L Liang W Xu T Jiang W. LncRNA RP11-361F15.2 promotes osteosarcoma tumorigenesis by inhibiting M2-like polarization of tumor-associated macrophages of CPEB4. Cancer Lett (2020) 473:33–49. doi: 10.1016/j.canlet.2019.12.041 31904478

[36] Gao Y Fang P Li W Zhang J Wang G Jiang D. LncRNA NEAT1 sponges miR-214 to regulate M2 macrophage polarization by regulation of B7-H3 in multiple myeloma. Mol Immunol (2020) 117:20–8. doi: 10.1016/j.molimm.2019.10.026 31731055

[37] Tao S Chen Q Lin C Dong H. Linc00514 promotes breast cancer metastasis and M2 polarization of tumor-associated macrophages *via* Jagged1-mediated notch signaling pathway. J Exp Clin Canc Res (2020) 39 :191. doi: 10.1186/s13046-020-01676-x PMC7500027 32943090

[38] Han C Yang Y Sheng Y Wang J Li W Zhou X. The mechanism of lncRNA-CRNDE in regulating tumour-associated macrophage M2 polarization and promoting tumour angiogenesis. J Cell Mol Med (2021) 25:4235–47. doi: 10.1111/jcmm.16477 PMC8093957 33742511

[39] Cao J Dong R Jiang L Gong Y Yuan M You J. LncRNA-MM2P identified as a modulator of macrophage m2 polarization. Cancer Immunol Res (2019) 7:292–305. doi: 10.1158/2326-6066.CIR-18-0145 30459152

[40] Zong S Dai W Guo X Wang K. LncRNA-SNHG1 promotes macrophage M2-like polarization and contributes to breast cancer growth and metastasis. Aging (Albany NY.) (2021) 13:23169–81. doi: 10.18632/aging.203609 PMC8544328 34618681

[41] Tian X Wu Y Yang Y Wang J Niu M Gao S. Long noncoding RNA LINC00662 promotes M2 macrophage polarization and hepatocellular carcinoma progression *via* activating wnt/beta-catenin signaling. Mol Oncol (2020) 14:462–83. doi: 10.1002/1878-0261.12606 PMC6998656 31785055

[42] Sun Y Xu J. TCF-4 regulated lncRNA-XIST promotes M2 polarization of macrophages and is associated with lung cancer. Onco Targets Ther (2019) 12:8055–62. doi: 10.2147/OTT.S210952 PMC6781636 31632059

[43] Ai Y Liu S Luo H Wu S Wei H Tang Z. LncRNA DCST1-AS1 facilitates oral squamous cell carcinoma by promoting m2 macrophage polarization through activating NF-κB signaling. J Immunol Res (2021) 2021:1–9. doi: 10.1155/2021/5524231 PMC8369177 34414241

[44] Zhou L Tian Y Guo F Yu B Li J Xu H. LincRNA-p21 knockdown reversed tumor-associated macrophages function by promoting MDM2 to antagonize* p53 activation and alleviate breast cancer development. Cancer Immunol Immunother (2020) 69:835–46. doi: 10.1007/s00262-020-02511-0 PMC11027865 32062693

[45] Chen C He W Huang J Wang B Li H Cai Q. LNMAT1 promotes lymphatic metastasis of bladder cancer *via* CCL2 dependent macrophage recruitment. Nat Commun (2018) 9 :3826. doi: 10.1038/s41467-018-06152-x 30237493 PMC6148066

[46] Wang Y Yu G Liu Y Xie L Tianxin Ge J Zhao G. Hypoxia-induced PTTG3P contributes to colorectal cancer glycolysis and M2 phenotype of macrophage. Bioscience Rep (2021) 41 :BSR20210764. doi: 10.1042/BSR20210764 PMC8264182 34132347

[47] Yang B Su K Sha G Bai Q Sun G Chen H. LINC00665 interacts with BACH1 to activate Wnt1 and mediates the M2 polarization of tumor-associated macrophages in GC. Mol Immunol (2022) 146:1–8. doi: 10.1016/j.molimm.2022.03.120 35395473

[48] Liu Y Shi M He X Cao Y Liu P Li F. LncRNA-PACERR induces pro-tumour macrophages *via* interacting with miR-671-3p and m6A-reader IGF2BP2 in pancreatic ductal adenocarcinoma. J Hematol Oncol (2022) 15:52. doi: 10.1186/s13045-022-01272-w 35526050 PMC9077921

[49] Liu Y Wang X Zhu Y Cao Y Wang L Li F. The CTCF/LncRNA-PACERR complex recruits E1A binding protein p300 to induce pro-tumour macrophages in pancreatic ductal adenocarcinoma *via* directly regulating PTGS2 expression. Clin Transl Med (2022) 12:e654. doi: 10.1002/ctm2.654 35184402 PMC8858628

[50] Xie C Guo Y Lou S. LncRNA ANCR promotes invasion and migration of gastric cancer by regulating FoxO1 expression to inhibit macrophage m1 polarization. Digest Dis Sci (2020) 65:2863–72. doi: 10.1007/s10620-019-06019-1 31894487

[51] Zhu L Liu Y Tang H Wang P. FOXP3 activated-LINC01232 accelerates the stemness of non-small cell lung carcinoma by activating TGF-beta signaling pathway and recruiting IGF2BP2 to stabilize TGFBR1. Exp Cell Res (2022) 413:113024. doi: 10.1016/j.yexcr.2022.113024 35026283

[52] Lai F Zhang H Xu B Xie Y Yu H. Long non-coding RNA NBR2 suppresses the progress of colorectal cancer *in vitro* and *in vivo* by regulating the polarization of TAM. Bioengineered (2021) 12:5462–75. doi: 10.1080/21655979.2021.1958558 PMC8806745 34506209

[53] Zhou Y Zhao W Mao L Wang Y Xia L Cao M. Long non-coding RNA NIFK-AS1 inhibits M2 polarization of macrophages in endometrial cancer through targeting miR-146a. Int J Biochem Cell Biol (2018) 104:25–33. doi: 10.1016/j.biocel.2018.08.017 30176290

[54] Ye Y Xu Y Lai Y He W Li Y Wang R. Long non-coding RNA cox-2 prevents immune evasion and metastasis of hepatocellular carcinoma by altering M1/M2 macrophage polarization. J Cell Biochem (2018) 119:2951–63. doi: 10.1002/jcb.26509 29131381

[55] Zhang Y Feng J Fu H Liu C Yu Z Sun Y. Coagulation factor x regulated by CASC2c recruited macrophages and induced m2 polarization in glioblastoma multiforme. Front Immunol (2018) 9:1557. doi: 10.3389/fimmu.2018.01557 30034397 PMC6043648

[56] Zhao Y Yu Z Ma R Zhang Y Zhao L Yan Y. LncRNA-Xist/miR-101-3p/KLF6/C/EBPalpha axis promotes TAM polarization to regulate cancer cell proliferation and migration. Mol Ther Nucleic Acids (2021) 23:2020.12.00510.3389/fimmu.2020.01731. doi: 10.1016/j.omtn.2020.12.00510.3389/fimmu.2020.01731 PMC7810606 33510942

[57] Zhou Z Wang Z Gao J Lin Z Wang Y Shan P. Noncoding RNA-mediated macrophage and cancer cell crosstalk in hepatocellular carcinoma. Mol Ther Oncolytics (2022) 25:98–120. doi: 10.1016/j.omto.2022.03.002 35506150 PMC9024380

[58] Cen L Liu R Liu W Li Q Cui H. Competing endogenous RNA networks in glioma. Front Genet (2021) 12:675498. doi: 10.3389/fgene.2021.675498 33995499 PMC8117106

[59] Abdollahzadeh R Daraei A Mansoori Y Sepahvand M Amoli MM Tavakkoly-Bazzaz J. Competing endogenous RNA (ceRNA) cross talk and language in ceRNA regulatory networks: A new look at hallmarks of breast cancer. J Cell Physiol (2019) 234:10080–100. doi: 10.1002/jcp.27941 30537129

[60] Zhao M Feng J Tang L. Competing endogenous RNAs in lung cancer. Cancer Biol Med (2021) 18:1–20. doi: 10.20892/j.issn.2095-3941.2020.0203 33628581 PMC7877185

[61] Ye J Li J Zhao P. Roles of ncRNAs as ceRNAs in gastric cancer. Genes (Basel) (2021) 12 :1036. doi: 10.3390/genes12071036 34356052 PMC8305186

[62] Han TS Hur K Cho HS Ban HS. Epigenetic associations between lncRNA/circRNA and miRNA in hepatocellular carcinoma. Cancers (Basel) (2020) 12 :2622. doi: 10.3390/cancers12092622 PMC7565033 32937886

[63] Wang L Cho KB Li Y Tao G Xie Z Guo B. Long noncoding RNA (lncRNA)-mediated competing endogenous RNA networks provide novel potential biomarkers and therapeutic targets for colorectal cancer. Int J Mol Sci (2019) 20 :5758. doi: 10.3390/ijms20225758 PMC6888455 31744051

[64] Xu J Xu J Liu X Jiang J. The role of lncRNA-mediated ceRNA regulatory networks in pancreatic cancer. Cell Death Discov (2022) 8:287. doi: 10.1038/s41420-022-01061-x 35697671 PMC9192730

[65] Yang Y Deng X Li Q Wang F Miao L Jiang Q. Emerging roles of long noncoding RNAs in cholangiocarcinoma: Advances and challenges. Cancer Commun (Lond) (2020) 40:655–80. doi: 10.1002/cac2.12109 PMC7743012 33142045

[66] Braga EA Fridman MV Moscovtsev AA Filippova EA Dmitriev AA Kushlinskii NE. LncRNAs in ovarian cancer progression, metastasis, and main pathways: CeRNA and alternative mechanisms. Int J Mol Sci (2020) 21 :8855. doi: 10.3390/ijms21228855 PMC7700431 33238475

[67] Bai Q Pan Z Nabi G Rashid F Liu Y Khan S. Emerging role of competing endogenous RNA and associated noncoding RNAs in thyroid cancer. Am J Cancer Res (2022) 12:961–73. PMC8984881 35411240

[68] Braga EA Fridman MV Filippova EA Loginov VI Pronina IV Burdennyy AM. LncRNAs in the regulation of genes and signaling pathways through miRNA-mediated and other mechanisms in clear cell renal cell carcinoma. Int J Mol Sci (2021) 22 :11193. doi: 10.3390/ijms222011193 34681854 PMC8539140

[69] Yang N Liu K Yang M Gao X. CeRNAs in cancer: Mechanism and functions in a comprehensive regulatory network. J Oncol (2021) 2021:4279039. doi: 10.1155/2021/4279039 34659409 PMC8516523

[70] Shao F Pang X Baeg GH. Targeting the JAK/STAT signaling pathway for breast cancer. Curr Med Chem (2021) 28:5137–51. doi: 10.2174/0929867328666201207202012 33290193

[71] Gutierrez-Hoya A Soto-Cruz I. Role of the JAK/STAT pathway in cervical cancer: Its relationship with HPV E6/E7 oncoproteins. Cells-Basel (2020) 9 :2297. doi: 10.3390/cells9102297 PMC7602614 33076315

[72] Ni Y Low JT Silke J O'Reilly LA. Digesting the role of JAK-STAT and cytokine signaling in oral and gastric cancers. Front Immunol (2022) 13:835997. doi: 10.3389/fimmu.2022.835997 35844493 PMC9277720

[73] Hin TJ Hao TD Lim JJ Toh TB. JAK/STAT signaling in hepatocellular carcinoma. Hepat Oncol (2020) 7:P18. doi: 10.2217/hep-2020-0001 PMC7137178 32273976

[74] Farooqi AA Nayyab S Martinelli C Berardi R Katifelis H Gazouli M. Regulation of hippo, TGFbeta/SMAD, wnt/beta-catenin, JAK/STAT, and NOTCH by long non-coding RNAs in pancreatic cancer. Front Oncol (2021) 11:657965. doi: 10.3389/fonc.2021.657965 34178644 PMC8220219

[75] Mohrherr J Uras IZ Moll HP Casanova E. STAT3: Versatile functions in non-small cell lung cancer. Cancers (Basel) (2020) 12 :1107. doi: 10.3390/cancers12051107 PMC7281271 32365499

[76] Ou A Ott M Fang D Heimberger AB. The role and therapeutic targeting of JAK/STAT signaling in glioblastoma. Cancers (Basel) (2021) 13 :437. doi: 10.3390/cancers13030437 33498872 PMC7865703

[77] Malekan M Ebrahimzadeh MA Sheida F. The role of hypoxia-inducible factor-1alpha and its signaling in melanoma. BioMed Pharmacother (2021) 141:111873. doi: 10.1016/j.biopha.2021.111873 34225012

[78] Raivola J Haikarainen T Abraham BG Silvennoinen O. Janus kinases in leukemia. Cancers (Basel) (2021) 13 :800. doi: 10.3390/cancers13040800 33672930 PMC7918039

[79] Waldmann TA. JAK/STAT pathway directed therapy of T-cell leukemia/lymphoma: Inspired by functional and structural genomics. Mol Cell Endocrinol (2017) 451:66–70. doi: 10.1016/j.mce.2017.02.019 28214593 PMC5469693

[80] O'Sullivan JM Harrison CN. JAK-STAT signaling in the therapeutic landscape of myeloproliferative neoplasms. Mol Cell Endocrinol (2017) 451:71–9. doi: 10.1016/j.mce.2017.01.050 28167129

[81] Krishnamurthy N Kurzrock R. Targeting the wnt/beta-catenin pathway in cancer: Update on effectors and inhibitors. Cancer Treat Rev (2018) 62:50–60. doi: 10.1016/j.ctrv.2017.11.002 29169144 PMC5745276

[82] Koni M Pinnaro V Brizzi MF. The wnt signalling pathway: A tailored target in cancer. Int J Mol Sci (2020) 21 :7697. doi: 10.3390/ijms21207697 PMC7589708 33080952

[83] Lalle G Twardowski J Grinberg-Bleyer Y. NF-kappaB in cancer immunity: Friend or foe? Cells-Basel (2021) 10 :355. doi: 10.3390/cells10020355 PMC7914614 33572260

[84] Fu LQ Du WL Cai MH Yao JY Zhao YY Mou XZ. The roles of tumor-associated macrophages in tumor angiogenesis and metastasis. Cell Immunol (2020) 353:104119. doi: 10.1016/j.cellimm.2020.104119 32446032

[85] Zeng X Xie H Yuan J Jiang X Yong J Zeng D. M2-like tumor-associated macrophages-secreted EGF promotes epithelial ovarian cancer metastasis *via* activating EGFR-ERK signaling and suppressing lncRNA LIMT expression. Cancer Biol Ther (2019) 20:956–66. doi: 10.1080/15384047.2018.1564567 PMC6606001 31062668

[86] Viallard C Larrivee B. Tumor angiogenesis and vascular normalization: Alternative therapeutic targets. Angiogenesis (2017) 20:409–26. doi: 10.1007/s10456-017-9562-9 28660302

[87] Dong F Ruan S Wang J Xia Y Le K Xiao X. M2 macrophage-induced lncRNA PCAT6 facilitates tumorigenesis and angiogenesis of triple-negative breast cancer through modulation of VEGFR2. Cell Death Dis (2020) 11:728. doi: 10.1038/s41419-020-02926-8 32908134 PMC7481779

[88] Zheng T Ma G Tang M Li Z Xu R. IL-8 secreted from M2 macrophages promoted prostate tumorigenesis *via* STAT3/MALAT1 pathway. Int J Mol Sci (2019) 20:98. doi: 10.3390/ijms20010098 PMC6337597 30591689

[89] Cardoso AP Pinto ML Castro F Costa AM Marques-Magalhaes A Canha-Borges A. The immunosuppressive and pro-tumor functions of CCL18 at the tumor microenvironment. Cytokine Growth Factor Rev (2021) 60:107–19. doi: 10.1016/j.cytogfr.2021.03.005 33863622

[90] Long L Hu Y Long T Lu X Tuo Y Li Y. Tumor-associated macrophages induced spheroid formation by CCL18-ZEB1-M-CSF feedback loop to promote transcoelomic metastasis of ovarian cancer. J Immunother Cancer (2021) 9 :e003973. doi: 10.1136/jitc-2021-003973 34969774 PMC8718465

[91] Su Y Zhou Y Sun Y Wang Y Yin J Huang Y. Macrophage-derived CCL18 promotes osteosarcoma proliferation and migration by upregulating the expression of UCA1. J Mol Med (2019) 97:49–61. doi: 10.1007/s00109-018-1711-0 30426155

[92] He F Ding G Jiang W Fan X Zhu L. Effect of tumor-associated macrophages on lncRNA PURPL/miR-363/PDZD2 axis in osteosarcoma cells. Cell Death Discov (2021) 7:307. doi: 10.1038/s41420-021-00700-z 34686652 PMC8536668

[93] Kalluri R LeBleu VS. The biology, function, and biomedical applications of exosomes. Science (2020) 367 :eaau6977. doi: 10.1126/science.aau6977 32029601 PMC7717626

[94] Fan Q Yang L Zhang X Peng X Wei S Su D. The emerging role of exosome-derived non-coding RNAs in cancer biology. Cancer Lett (2018) 414:107–15. doi: 10.1016/j.canlet.2017.10.040 29107112

[95] Naderi-Meshkin H Lai X Amirkhah R Vera J Rasko JEJ Schmitz U. Exosomal lncRNAs and cancer: Connecting the missing links. Bioinformatics (2019) 35:352–60. doi: 10.1093/bioinformatics/bty527 30649349

[96] Kok VC Yu CC. Cancer-derived exosomes: Their role in cancer biology and biomarker development. Int J Nanomedicine (2020) 15:8019–36. doi: 10.2147/IJN.S272378 PMC7585279 33116515

[97] Yousefi H Maheronnaghsh M Molaei F Mashouri L Reza AA Momeny M. Long noncoding RNAs and exosomal lncRNAs: Classification, and mechanisms in breast cancer metastasis and drug resistance. Oncogene (2020) 39:953–74. doi: 10.1038/s41388-019-1040-y 31601996

[98] Chen Q Li Y Liu Y Xu W Zhu X. Exosomal non-coding RNAs-mediated crosstalk in the tumor microenvironment. Front Cell Dev Biol (2021) 9:646864. doi: 10.3389/fcell.2021.646864 33912560 PMC8072401

[99] Robless EE Howard JA Casari I Falasca M. Exosomal long non-coding RNAs in the diagnosis and oncogenesis of pancreatic cancer. Cancer Lett (2021) 501:55–65. doi: 10.1016/j.canlet.2020.12.005 33359452

[100] Pathania AS Challagundla KB. Exosomal long non-coding RNAs: Emerging players in the tumor microenvironment. Mol Ther Nucleic Acids (2021) 23:1371–83. doi: 10.1016/j.omtn.2020.09.039 PMC7940039 33738133

[101] Wang Y Zhang M Zhou F. Biological functions and clinical applications of exosomal long non-coding RNAs in cancer. J Cell Mol Med (2020) 24:11656–66. doi: 10.1111/jcmm.15873 PMC7578871 32924276

[102] Wang M Zhou L Yu F Zhang Y Li P Wang K. The functional roles of exosomal long non-coding RNAs in cancer. Cell Mol Life Sci (2019) 76:2059–76. doi: 10.1007/s00018-019-03018-3 PMC11105177 30683984

[103] Zheng R Du M Wang X Xu W Liang J Wang W. Exosome-transmitted long non-coding RNA PTENP1 suppresses bladder cancer progression. Mol Cancer (2018) 17:143. doi: 10.1186/s12943-018-0880-3 30285771 PMC6169076

[104] Chen C Luo Y He W Zhao Y Kong Y Liu H. Exosomal long noncoding RNA LNMAT2 promotes lymphatic metastasis in bladder cancer. J Clin Invest (2020) 130:404–21. doi: 10.1172/JCI130892 PMC6934220 31593555

[105] Lin LY Yang L Zeng Q Wang L Chen ML Zhao ZH. Tumor-originated exosomal lncUEGC1 as a circulating biomarker for early-stage gastric cancer. Mol Cancer (2018) 17:84. doi: 10.1186/s12943-018-0834-9 29690888 PMC5978993

[106] Li W Zhang L Guo B Deng J Wu S Li F. Exosomal FMR1-AS1 facilitates maintaining cancer stem-like cell dynamic equilibrium *via* TLR7/NFkappaB/c-myc signaling in female esophageal carcinoma. Mol Cancer (2019) 18:22. doi: 10.1186/s12943-019-0949-7 30736860 PMC6367809

[107] Maia J Caja S Strano Moraes MC Couto N Costa-Silva B. Exosome-based cell-cell communication in the tumor microenvironment. Front Cell Dev Biol (2018) 6:18. doi: 10.3389/fcell.2018.00018 29515996 PMC5826063

[108] Xin L Wu Y Liu C Zeng F Wang J Wu D. Exosome-mediated transfer of lncRNA HCG18 promotes M2 macrophage polarization in gastric cancer. Mol Immunol (2021) 140:196–205. doi: 10.1016/j.molimm.2021.10.011 34735868

[109] Yao H Tian L Yan B Yang L Li Y. LncRNA TP73-AS1 promotes nasopharyngeal carcinoma progression through targeting miR-342-3p and M2 polarization *via* exosomes. Cancer Cell Int (2022) 22 :16. doi: 10.1186/s12935-021-02418-5 35012518 PMC8751349

[110] Zhou D Xia Z Xie M Gao Y Yu Q He B. Exosomal long non-coding RNA SOX2 overlapping transcript enhances the resistance to EGFR-TKIs in non-small cell lung cancer cell line H1975. Hum Cell (2021) 34:1478–89. doi: 10.1007/s13577-021-00572-6 34244990

[111] Wang LP Lin J Ma XQ Xu DY Shi CF Wang W. Exosomal DLX6-AS1 from hepatocellular carcinoma cells induces M2 macrophage polarization to promote migration and invasion in hepatocellular carcinoma through microRNA-15a-5p/CXCL17 axis. J Exp Clin Cancer Res (2021) 40:177. doi: 10.1186/s13046-021-01973-z 34039401 PMC8152341

[112] Li X Lei Y Wu M Li N. Regulation of macrophage activation and polarization by HCC-derived exosomal lncRNA TUC339. Int J Mol Sci (2018) 19:2958. doi: 10.3390/ijms19102958 PMC6213212 30274167

[113] Liang Z Liu H Wang F Xiong L Zhou C Hu T. LncRNA RPPH1 promotes colorectal cancer metastasis by interacting with TUBB3 and by promoting exosomes-mediated macrophage M2 polarization. Cell Death Dis (2019) 10 :829. doi: 10.1038/s41419-019-2077-0 31685807 PMC6828701

[114] Jiang H Zhou L Shen N Ning X Wu D Jiang K. M1 macrophage-derived exosomes and their key molecule lncRNA HOTTIP suppress head and neck squamous cell carcinoma progression by upregulating the TLR5/NF-κB pathway. Cell Death Dis (2022) 13:183. doi: 10.1038/s41419-022-04640-z 35210436 PMC8873565

[115] Liu Y Lin W Dong Y Li X Lin Z Jia J. Long noncoding RNA HCG18 up-regulates the expression of WIPF1 and YAP/TAZ by inhibiting miR-141-3p in gastric cancer. Cancer Med (2020) 9:6752–65. doi: 10.1002/cam4.3288 PMC7520348 32725768

[116] Zhang F Luo BH Wu QH Li QL Yang KD. LncRNA HCG18 upregulates TRAF4/TRAF5 to facilitate proliferation, migration and EMT of epithelial ovarian cancer by targeting miR-29a/b. Mol Med (2022) 28:2. doi: 10.1186/s10020-021-00415-y 34983361 PMC8725507

[117] Mi X Xu R Hong S Xu T Zhang W Liu M. M2 macrophage-derived exosomal lncRNA AFAP1-AS1 and MicroRNA-26a affect cell migration and metastasis in esophageal cancer. Mol Ther - Nucleic Acids (2020) 22:779–90. doi: 10.1016/j.omtn.2020.09.035 PMC7595846 33230475

[118] Zhang H Yu Y Wang J Han Y Ren T Huang Y. Macrophages-derived exosomal lncRNA LIFR-AS1 promotes osteosarcoma cell progression *via* miR-29a/NFIA axis. Cancer Cell Int (2021) 21 :192. doi: 10.1186/s12935-021-01893-0 33794884 PMC8017664

[119] Xin L Zhou LQ Liu C Zeng F Yuan YW Zhou Q. Transfer of LncRNA CRNDE in TAM-derived exosomes is linked with cisplatin resistance in gastric cancer. EMBO Rep (2021) 22:e52124. doi: 10.15252/embr.202052124 34647680 PMC8647143

[120] Wu Q Wu X Ying X Zhu Q Wang X Jiang L. Suppression of endothelial cell migration by tumor associated macrophage-derived exosomes is reversed by epithelial ovarian cancer exosomal lncRNA. Cancer Cell Int (2017) 17 :62. doi: 10.1186/s12935-017-0430-x 28592924 PMC5461704

[121] Chen J Zhang K Zhi Y Wu Y Chen B Bai J. Tumor-derived exosomal miR-19b-3p facilitates M2 macrophage polarization and exosomal LINC00273 secretion to promote lung adenocarcinoma metastasis *via* hippo pathway. Clin Trans Med (2021) 11 :e478. doi: 10.1002/ctm2.478 PMC8435259 34586722

[122] Rinaldi C Wood M. Antisense oligonucleotides: The next frontier for treatment of neurological disorders. Nat Rev Neurol (2018) 14:9–21. doi: 10.1038/nrneurol.2017.148 29192260

[123] Li M Ding X Zhang Y Li X Zhou H Yang L. Antisense oligonucleotides targeting lncRNA AC104041.1 induces antitumor activity through Wnt2B/beta-catenin pathway in head and neck squamous cell carcinomas. Cell Death Dis (2020) 11:672. doi: 10.1038/s41419-020-02820-3 32826863 PMC7443144

[124] Vidovic D Huynh TT Konda P Dean C Cruickshank BM Sultan M. ALDH1A3-regulated long non-coding RNA NRAD1 is a potential novel target for triple-negative breast tumors and cancer stem cells. Cell Death Differ (2020) 27:363–78. doi: 10.1038/s41418-019-0362-1 PMC7206030 31197235

[125] Jin X Xu XE Jiang YZ Liu YR Sun W Guo YJ. The endogenous retrovirus-derived long noncoding RNA TROJAN promotes triple-negative breast cancer progression *via* ZMYND8 degradation. Sci Adv (2019) 5:t9820. doi: 10.1126/sciadv.aat9820 PMC6402854 30854423

[126] Liu QP Lin JY An P Chen YY Luan X Zhang H. LncRNAs in tumor microenvironment: The potential target for cancer treatment with natural compounds and chemical drugs. Biochem Pharmacol (2021) 193:114802. doi: 10.1016/j.bcp.2021.114802 34678226

[127] Wang X Wang X Xu M Sheng W. Emerging roles of long noncoding RNAs in immuno-oncology. Front Cell Dev Biol (2021) 9:722904. doi: 10.3389/fcell.2021.722904 34900986 PMC8655840

[128] Li S Yi M Dong B Tan X Luo S Wu K. The role of exosomes in liquid biopsy for cancer diagnosis and prognosis prediction. Int J Cancer (2021) 148:2640–51. doi: 10.1002/ijc.33386 PMC8049049 33180334

[129] Tovar-Camargo OA Toden S Goel A. Exosomal microRNA biomarkers: Emerging frontiers in colorectal and other human cancers. Expert Rev Mol Diagn (2016) 16:553–67. doi: 10.1586/14737159.2016.1156535 PMC4935983 26892862

[130] Li MY Liu LZ Dong M. Progress on pivotal role and application of exosome in lung cancer carcinogenesis, diagnosis, therapy and prognosis. Mol Cancer (2021) 20:22. doi: 10.1186/s12943-021-01312-y 33504342 PMC7839206

[131] Wang M Ji S Shao G Zhang J Zhao K Wang Z. Effect of exosome biomarkers for diagnosis and prognosis of breast cancer patients. Clin Transl Oncol (2018) 20:906–11. doi: 10.1007/s12094-017-1805-0 29143228

[132] Wu H Fu M Liu J Chong W Fang Z Du F. The role and application of small extracellular vesicles in gastric cancer. Mol Cancer (2021) 20:71. doi: 10.1186/s12943-021-01365-z 33926452 PMC8081769

[133] Sasaki R Kanda T Yokosuka O Kato N Matsuoka S Moriyama M. Exosomes and hepatocellular carcinoma: From bench to bedside. Int J Mol Sci (2019) 20 :1406. doi: 10.3390/ijms20061406 PMC6471845 30897788

[134] Xiao Y Zhong J Zhong B Huang J Jiang L Jiang Y. Exosomes as potential sources of biomarkers in colorectal cancer. Cancer Lett (2020) 476:13–22. doi: 10.1016/j.canlet.2020.01.033 32044357

[135] Wu H Chen X Ji J Zhou R Liu J Ni W. Progress of exosomes in the diagnosis and treatment of pancreatic cancer. Genet Test Mol Biomarkers (2019) 23:215–22. doi: 10.1089/gtmb.2018.0235 30793953

[136] Lorenc T Klimczyk K Michalczewska I Slomka M Kubiak-Tomaszewska G Olejarz W. Exosomes in prostate cancer diagnosis, prognosis and therapy. Int J Mol Sci (2020) 21 :2118. doi: 10.3390/ijms21062118 PMC7139716 32204455

[137] Zhang W Ou X Wu X. Proteomics profiling of plasma exosomes in epithelial ovarian cancer: A potential role in the coagulation cascade, diagnosis and prognosis. Int J Oncol (2019) 54:1719–33. doi: 10.3892/ijo.2019.4742 PMC6438431 30864689

[138] Hofmann L Ludwig S Vahl JM Brunner C Hoffmann TK Theodoraki MN. The emerging role of exosomes in diagnosis, prognosis, and therapy in head and neck cancer. Int J Mol Sci (2020) 21 :4072. doi: 10.3390/ijms21114072 PMC7312915 32517240

[139] Shi J Zhang Y Yao B Sun P Hao Y Piao H. Role of exosomes in the progression, diagnosis, and treatment of gliomas. Med Sci Monit (2020) 26:e924023. doi: 10.12659/MSM.924023 33245712 PMC7706139

[140] Pritchard A Tousif S Wang Y Hough K Khan S Strenkowski J. Lung tumor cell-derived exosomes promote m2 macrophage polarization. Cells-Basel (2020) 9 :1303. doi: 10.3390/cells9051303 PMC7290460 32456301

[141] Yuan X Qian N Ling S Li Y Sun W Li J. Breast cancer exosomes contribute to pre-metastatic niche formation and promote bone metastasis of tumor cells. Theranostics (2021) 11:1429–45. doi: 10.7150/thno.45351 PMC7738874 33391543

